# Electroacupuncture at Bilateral ST36 Acupoints: Inducing the Hypoglycemic Effect through Enhancing Insulin Signal Proteins in a Streptozotocin-Induced Rat Model during Isoflurane Anesthesia

**DOI:** 10.1155/2021/5852599

**Published:** 2021-10-06

**Authors:** Kee-Ming Man, Yu-Chen Lee, Ying-I. Chen, Yung Hsiang Chen, Shih Liang Chang, Chieh-Chen Huang

**Affiliations:** ^1^Department of Life Sciences, National Chung Hsing University, Taichung, Taiwan; ^2^Department of Medicinal Botanicals and Health Applications, College of Biotechnology and Bioresources, Da Yeh University, Changhua, Taiwan; ^3^Department of Anesthesiology, China Medical University Hsinchu Hospital, Hsinchu, Zhubei, Taiwan; ^4^Department of Acupuncture, China Medical University Hospital, Taichung, Taiwan; ^5^College of Chinese Medicine, Graduate Institute of Acupuncture Science, China Medical University, Taichung, Taiwan; ^6^Graduate Institute of Integrated Medicine, College of Chinese Medicine, China Medical University, Taichung, Taiwan; ^7^Department of Psychology, College of Medical and Health Science, Asia University, Taichung, Taiwan

## Abstract

In rats with 2-deoxy-2-(3-(methyl-3-nitrosoureido)-d-glucopyranose streptozotocin- (STZ-) induced insulin-dependent diabetes (IDDM), continuous 15 Hz electrical stimulation at bilateral ST36 acupoints for 30 and 60 minutes has been shown to prevent hyperglycemia. We hypothesized that the mechanism of action in STZ-induced IDDM rats is that electrical stimulation at bilateral ST36 acupoints is effective in improving insulin receptor substrate type 1 (IRS-1) and glucose transporter type 4 (GLUT4) protein expressions associated with counteracting both plasma glucose and free fatty acid (FFA) levels during isoflurane anesthesia. In this study, twenty-six healthy male Wistar rats, weighing 250–350 g and aged 8–10 weeks were tested. Rats in the experimental electroacupuncture (EA) group (*n* = 13) received 15 Hz electrical stimulation at bilateral ST 36 acupoints for 30 and 60 minutes. Rats in the control group (*n* = 13) were handled but not subjected to the stimulation treatment. In both IDDM and normal Wistar rats, we observed a negative change in plasma glucose levels when rats were given the EA treatment, but a positive change in plasma glucose without EA treatment relative to baseline. Within the IDDM group, a negative change in FFA levels was observed when rats were given the EA treatment, while a positive change in the FFA level was shown without the EA treatment. In the expressed protein signals, we found a significant elevation in both GLUT4 and IRS-1 proteins in the IDDM group treated by EA. Moreover, we found a significant mean difference between GLUT4 and IRS-1 protein expression levels relative to *β*-actin. Our findings suggested that EA at bilateral ST36 acupoints could serve as an effective strategy for lowering plasma glucose by decreasing free fatty acid levels and improving the expression of IRS-1 and GLUT4 proteins in a STZ-IDDM rat model during isoflurane anesthesia.

## 1. Introduction

In perioperative medicine practice, physiologic changes that occur in the hyperglycemic state are one of the risk factors that contribute to poor postoperative outcomes [1, 2]. However, the underlying mechanism(s) relating hyperglycemia to poor outcomes is not completely understood. Pathophysiologic changes resulting from elevated plasma glucose include impaired neutrophil function and cause an overproduction of reactive oxygen species, free fatty acids (FFA), and inflammatory mediators. These factors contribute to direct cellular damage and vascular and immune dysfunction and are associated with significantly infectious complications [3, 4]. Increased hospital length of stay, morbidity, and mortality after surgery are also associated with perioperative hyperglycemia [5]. Existing evidence indicates that correction of hyperglycemia with insulin therapy reduces hospital complications and decreases mortality in cardiac [6] and general surgery [7] patients.

Hyperglycemic reaction to surgical stress occurs not only more often [8] but is also prominent in diabetic patients during inhalation anesthesia. Isoflurane is the most widely used fluorinated ether inhalation anesthetic for the induction and maintenance of general anesthesia, where it can provide amnesic, analgesic, and muscle relaxant effects and attenuate the autonomic nervous system response to surgical stress. However, the depth of inhalation anesthesia and even deep anesthesia with inhalation anesthetics has no effect in preventing hyperglycemia during surgery [9]. In addition, several studies have shown that volatile anesthetics, such as isoflurane, impair adenosine triphosphate-sensitive potassium channel activity in pancreatic *β*-cells. This impairs insulin secretion [10, 11] and glucose utilization [11–13], increases target organ resistance to insulin [12, 14, 15], and results in hyperglycemia even without surgical stress [15–17].

In traditional Chinese medicine (TCM), acupuncture is applied in the treatment of diabetes mellitus. Electroacupuncture (EA) is a variant of regular acupuncture in which a small electric current is passed between pairs of acupuncture needles. The mechanisms of the hypoglycemic effect of EA have been clarified such that EA at specific acupoints increases *β*-endorphin secretion, which stimulates the secretion of insulin, in an insulin resistance rat model [18–20]. The underlying mechanism by which EA at specific acupoints reduces plasma glucose concentration has been shown to involve not only the stimulation of cholinergic nerves but also the adrenal glands in the streptozotocin- (STZ-) induced IDDM rat [21]. Furthermore, EA stimulation at different acupoints or with different frequencies affects hypoglycemic activity. Stimulation at 2 or 15 Hz on the same acupoints has been shown to target different mechanisms, namely, beta-endorphin from the adrenal gland by 2 Hz EA stimulation lowers plasma glucose [19], whereas multiple sources of endogenous opioid peptides are promoted by 15 Hz EA stimulation [20]. Finally, EA stimulation on bilateral ST36 acupoints with a higher hypoglycemic effect is mediated by the involvement of neurotransmitters, such as serotonin [22].

Insulin stimulates a signaling cascade that included activation of the insulin receptor (IR) and the serine phosphorylation of IR substrates (IRSs), such as IRS-1 [23]. This alters downstream signaling via phosphatidylinositol-3 kinase (PI3K) [24–26] and influences Akt (protein kinase B), thus playing a key role in regulating the translocation of the glucose transporter (GLUT4) from intracellular stores to the plasma membrane in muscle and adipose cells [27, 28]. Isoflurane anesthesia is reported to induce hyperglycemia associated with disturbance of insulin receptors and their related insulin signaling pathways in peripheral tissue, such as skeletal muscle and white adipocyte [29]. Although several previous studies revealed that the glucose-lowering effect of EA at specific acupoints in animal models contributes to insulin signaling pathways [30, 31], the present study not only aimed to explore the effect of EA on lowering plasma glucose but also to assess the microarray analysis of the effect of EA on the active insulin signaling pathway in a STZ-induced rat model of diabetes when subjected to exposure of isoflurane anesthesia.

## 2. Methods

We followed the general methodological approach of Tzeng et al. [32]. Differences in methods specific to our study are documented in this study.

### 2.1. Experimental Animals

Twenty-six healthy male Wistar rats, weighing 250–350 g and aged 8–10 weeks, were exposed to a regular 12-hour dark/light cycle (light on at 0700 h and off at 1900 h) and kept two per cage at 22 ± 2°C. The animals had access to food and water ad libitum. To establish the insulin-dependent diabetic (IDDM) model, rats (*n* = 18) were induced by femoral intravenous administration of 60 mg/kg streptozotocin (STZ) after they had fasted for 3 days. Glucose levels were checked to confirm the STZ-induced IDDM model before running the experiments. Consistent with previous research, if the blood glucose level was greater than 350 mg/dL, the rats were included in the STZ-induced IDDM model [18, 33]. For all subsequent experimental manipulations using fasted rats, food was withdrawn for 16 hours (from 16:30 h to 08:30 h) before the start of the experiment. All experimental procedures were conducted in accordance with Committee's Guidelines and Regulations for Animal Care and were approved by the Animal Care and Use Committee of the University of DYU. The study protocol was approved by the Institutional Animal Care and Use Committee (IACUC) of Da-Yeh University, Changhua, Taiwan (reference no. 105037). All experiments were carried out in accordance with the National Institutes of Health “Guide for the Care and Use of Laboratory Animals.”

### 2.2. Study Design

#### 2.2.1. Experiment 1

Normal Wistar rats were randomly allocated to the EA group (*n* = 4) or non-EA group (*n* = 4) as controls. After fasting 16 hours, initially, general anesthesia was induced by 5% isoflurane in oxygen and isoflurane was decreased to 1.5% in mixed with oxygen for maintenance of general anesthesia throughout the duration of the procedure.

#### 2.2.2. Experiment 2

IDDM rats were randomly allocated to the EA group (*n* = 9) or non-EA group (*n* = 9). After fasting 16 hours, initially, general anesthesia was also induced by 5% isoflurane in oxygen and isoflurane was decreased to 1.5% in mixed with oxygen for maintenance of the anesthesia throughout the duration of procedure. We collected the blood at 0, 30, and 60 minutes before and after EA either in the EA group or non-EA group. The total operation time was 1 hour, and isoflurane was removed after treatment.

The distribution of Wistar rats across experimental groupings above is given in Table 1.

### 2.3. Electroacupuncture

**Figure 1:**
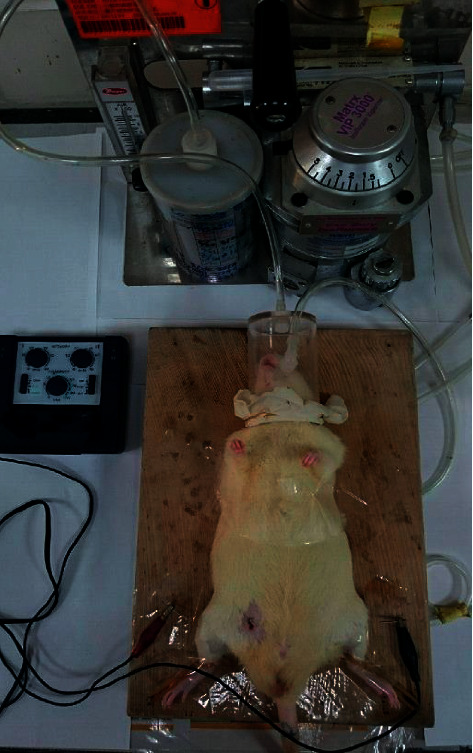
Locations of bilateral ST36 acupoints in rats. Brown represents the positive charge and black represents the negative charge connected to the EA instrument.

Electroacupuncture (EA) was performed by needling perpendicularly to 5 mm depth with 1.3 cm 32-gauge stainless steel acupuncture needles (Chian Huei Ltd., Taiwan) at the bilateral ST36 acupuncture points, which are located on the tibialis anterior muscle around the upper 1/6 of the length of the lower leg below the knee and based on the measurement of body length (Figure 1), as described previously [10], for 30 minutes and 60 minutes with 15 Hz frequency using a HANS LY257 stimulator (Healthtronics, Singapore).

### 2.4. Measurement of Plasma Glucose and FFA

We collected blood from both groups under isoflurane anesthesia. As a baseline for both groups, 0.3–0.5 mL of venous blood was drawn from the femoral vein by a 1 ml syringe at 0, 30, and 60 minutes of isoflurane anesthesia. The blood was placed in the Eppendorf tube and stored on ice with gentle shaking before centrifuging at 15,000 rpm for 5 minutes for testing plasma glucose and FFA. Plasma glucose levels (mg/dL) and FFA levels (mEq/L) were quantified using the Roche automated system and nonesterified fatty acid kits (Randox Laboratories Ltd., Canada), respectively, and read using an automatic spectrophotometer (COBAS system). The hypoglycemic effect (%) was calculated as

(1) $$\begin{array}{c} \frac{{\text{PG}}_{30,60\min}-{\text{PG}}_{0\min}}{{\text{PG}}_{0\min}}\cdot 100\%, \end{array}$$

where PG denotes the measured plasma glucose level and the subscript denotes the time in which it was measured (at 30 min, 60 min, or immediately (0 min)) [33].

### 2.5. Euthanasia, Tissue Sampling, and Western Blot Analysis

The rats were euthanized by using carbon dioxide at the end of experiment. For completeness, we recapitulate the harvested gastrocnemius muscles procedures and Western Blot analysis for the detection and expression of IRS-1 and GLUT4 proteins in gastrocnemius muscles as previously described [32, 34–36]. The harvested gastrocnemius muscles were weighed and washed with precold PBS before being lysed in radioimmunoprecipitation assay (RIPA) lysis buffer and ground for total protein extraction. After extraction, the proteins were separated by gel electrophoresis and transferred to membranes for incubation with specific antibodies [34–36]. The expression of insulin-signaling proteins, IRS-1 and GLUT4, was quantified relative to the expression of *β*-actin [34–36].

### 2.6. Statistical Analysis

To examine the effect of electroacupuncture (EA) manipulation on both plasma glucose and FFA levels across our experimental groups, we specified a random effects general linear model (i.e., mixed-effects GLM) that modeled the interaction between the rat group identity (IDDM or normal), experimental condition (EA or no-EA), and time (30 or 60 minutes after baseline). The three-way interaction and all nested two-way interactions and main effects were specified as fixed effects. The rat identity was included as a random intercept to model individual differences in plasma glucose and FFA levels across rats. Type III sum of squares for allowing the fixed-effects regressors to compete for variance were specified. We chose to use random effects GLM instead of the similar repeated-measures ANOVA approach given unequal datapoints across the measures; this approach allowed for more statistical power. We specified a similar model when looking at the effect of EA on protein signal levels, for both the GLUT4 and IRS-1 proteins. Again, we used random effects general linear model, this time modeling the interaction between the experimental condition (EA or non-EA) and the protein type (GLUT4 or IRS-1). Since protein signal levels were measured at the same time point and only within the IDDM group condition, these variables were not modeled here. Again, the two-way interaction and all nested main effects were specified as fixed effects, with rat identity included as a random intercept. For all analyses, a two-sided *p* < 0.05 was considered to represent statistical significance.

## 3. Results

Descriptive statistics for each measured variable across all rat identity groups and experimental treatment groups are reported in Table 2.

### 3.1. Plasma Glucose

**Figure 2:**
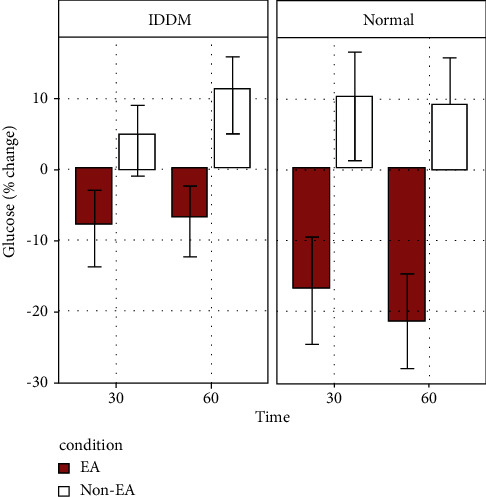
Serial plasma concentrations of glucose change (in percentage) in STZ-induced IDDM (left) and normal Wistar (right) rats treated with 15 Hz EA at bilateral ST36 acupoints (IDDM + EA group, *n* = 9; normal Wistar + EA group, *n* = 4) for 30 and 60 minutes or untreated (IDDM non-EA group, *n* = 9; normal Wistar non-EA group, *n* = 4) during isoflurane anesthesia.

A significant main effect of EA on plasma glucose levels was found (*F* (1, 24) = 29.472, *p* < 0.001; Figure 2). In both the IDDM and the normal Wistar groups, a negative change in plasma glucose levels relative to baseline was observed when rats were given EA treatment. On the other hand, a positive change in plasma glucose was found both in the IDDM group and the normal Wistar rat group without EA treatment. Furthermore, we did not find any significant effect of group or time nor did we find interactions between the effect of EA and any other variables.

### 3.2. Free Fatty Acid (FFA)

**Figure 3:**
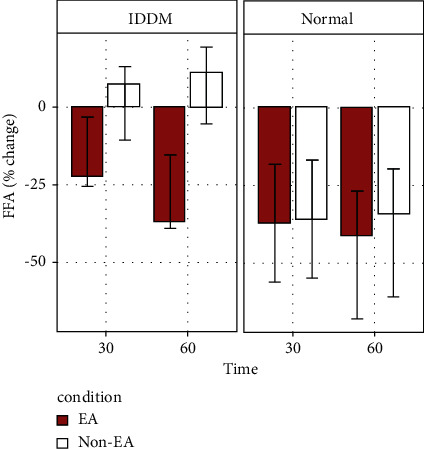
Serial plasma concentrations of free fatty acids (FFA) in STZ-induced IDDM (left) and normal Wistar (right) rats treated with 15 Hz EA at bilateral ST36 acupoints for 30 and 60 minutes or untreated during isoflurane anesthesia.

Similarly, a significant effect of EA on FFA levels (*F* (1, 17) = 4.964, *p*=0.039; Figure 3) was obtained. Within the IDDM group, a negative change relative to baseline in FFA levels was observed when rats were given the EA treatment. However, a positive change was found in FFA without EA treatment. Although we did not find a significant two-way interaction between EA condition and the rat group (*F* (1, 17) = 2.521, *p*=0.131), it was shown that the relative changes in FFA levels relative to baseline were only observed in the IDDM rats. All normal Wistar rats exhibited a negative change in FFA levels relative to baseline regardless of time or EA treatment (Figure 3).

### 3.3. Protein Expressions of GLUT4 and IRS-1

**Figure 4:**
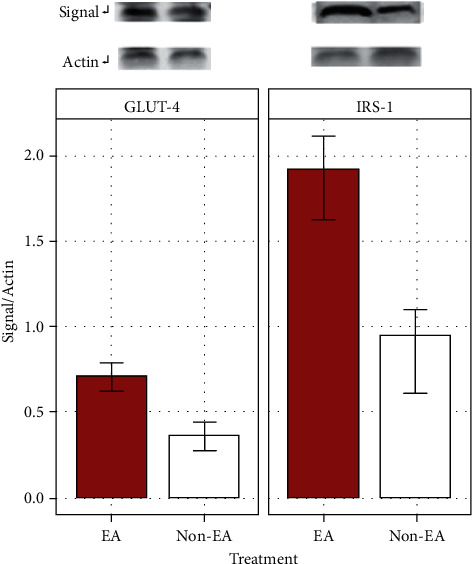
Protein expressions of insulin receptor substrate type 1 (IRS-1) and glucose transporter type 4 (GLUT4) relative to *β*-actin, measured by using Western blotting in skeletal muscle, received 15 Hz EA at bilateral ST36 acupoints for 30 and 60 minutes and untreated (white bar) during isoflurane anesthesia.

Increase in the protein expressions of GLUT4 and IRS-1 in the EA treatment group was observed when compared to *β*-actin (*F* (1, 20) = 12.596, *p*=0.002; Figure 4). Moreover, a significant mean difference between GLUT4 and IRS-1 protein expression was found when they were compared to *β*-actin (*F* (1, 20) = 23.286, *p*=0.0001).

## 4. Discussion

Substantial lines of research have demonstrated the effect of EA on hypoglycemic activity [33, 37, 38], and stimulation at specific acupoints also has been shown to be an effective method of managing the plasma glucose level during inhalation anesthesia [38]. The present study attempted to clarify the effect of EA on attenuating isoflurane-induced hyperglycemia by using insulin-dependent diabetic (IDDM) model rats, established by femoral intravenous administration of 60 mg/kg streptozotocin (STZ), after they had been fasted for 3 days. We demonstrated that EA with 15 Hz at bilateral ST36 acupoints for 30 minutes and 60 minutes in STZ-induced rats promoted a plasma glucose-lowering effect during isoflurane anesthesia, reflected by the significantly different changes in plasma glucose levels, when compared to the non-EA group. Although we did not find any significant hypoglycemic effect between the groups of IDDM and normal Wistar rats, the hypoglycemic effect was also significant in the group of normal Wistar rats treated with 15 Hz EA at ST36 for 30 minutes and 60 minutes during isoflurane anesthesia.

It is known that the fat metabolism produces FFA, which competes with IRS-1 to transport glucose into the cell in the oxidation pathway [39]. In addition, the elevated circulating free fatty acids affect both pancreatic beta-cell insulin secretion function which induces insulin deficiency [40, 41] and impair peripheral insulin sensitivity [42]. Furthermore, dose-dependent inhibition of insulin-stimulated glucose uptake and utilization occur with increasing free fatty acids [43]. Therefore, lowering plasma FFA to improve glucose utilization is an important issue [44]. Our study first replicated previous animal studies [17, 37] by showing that normal Wistar rats' FFA level were suppressed during isoflurane anesthesia, regardless of the EA vs. non-EA treatments. Importantly, our study extended this finding by showing that FFA is significantly decreased in STZ-induced IDDM rats treated with 15 Hz EA at bilateral ST36 for 30 minutes and 60 minutes, respectively, during isoflurane anesthesia. Together, these results show an important and differential impact of the EA manipulation between the STZ-induced IDDM and normal Wistar rat groups in terms of FFA change.

We also found a significantly increased expression of protein signals for both GLUT4 and IRS-1 proteins when they were compared to *β*-actin. In the EA group, we observed a significant increase of IRS-1 protein expression, indicating that EA may induce a plasma glucose-lowering effect in STZ-induced diabetic rats by activating IRS-1 and upregulating GLUT4 translocation from the cytosol to the cell membrane during isoflurane anesthesia. Glucose transporter 4 (GLUT4) is predominantly present in skeletal muscle, myocardium, and adipose tissue and is a major insulin-responding glucose transporter. In the absence of insulin, GLUT4 is found in GLUT4 storage vesicles within resting muscle and adipose cells, with less than 5% of the transporters present on the cell membranes, which can be isolated from cells and are found to contain numerous proteins in addition to GLUT4 [45]. The plasma membrane translocation of GLUT4 requires the intersection of insulin signaling and GLUT4 storage vesicles trafficking pathways. Our findings indicated that during isoflurane anesthesia, the hypoglycemic effect in STZ rats is mediated by EA stimulation at bilateral ST36 acupoints through enhancing the signal proteins, GLUT4 and IRS-1. However, the present study did not investigate whether EA enhanced the GLUT4 storage vesicles (GSVs) trafficking processes involved in the hypoglycemic activity of EA.

Enzymes that play an important role in the glucose metabolism [46], glucose homeostasis, and insulin signaling include plasma dipeptidyl peptidase-4 (DPP-4) and protein tyrosine phosphatase 1B (PTP-1B). DPP-4 increases in rats after STZ injection and positively correlates with blood glucose levels, thus playing a critical role in insulin secretion [47]; PTP-1B affects not only the upstream of the phosphatidylinositol 3-kinase pathway but also involves the dephosphorylation of insulin receptor and insulin receptor substrates [48]. The increased activities of DPP-4 and PTP-1B are associated with the occurrence of insulin resistance and diabetes. Therefore, future research is needed to determine whether EA improves the glucose metabolism and induces an insulin-sensitizing effect during inhalation anesthesia by inhibiting DPP-4 and PTP-1B activities.

Although isoflurane anesthesia could disturb insulin receptors and alter their related insulin-dependent signaling pathways in the liver, muscle, and adipose tissue [49], we only tested for the expression of IRS-1 and GLUT4. Other key proteins involved in insulin signaling downstream transduction would be investigated in STZ-induced diabetic rats during exposure to isoflurane in the future.

There is an increasing trend to use the combination of EA and oral hypoglycemic agents (OHA) for controlling glucose and improving insulin sensitivity in diabetic patients [50, 51]. However, volatile anesthetics, such as isoflurane and sevoflurane, induced glucose dyshomeostasis by affecting insulin. The current work suggests that EA overcomes the glucose metabolism dyshomeostasis induced by volatile anesthetics during anesthesia, probably by enhancing the expression of these proteins, IRS-1 and GLUT4. Follow-up work is needed to examine the effect of EA on the glucose metabolism specifically in the PI3K/Akt pathway during inhalation anesthesia.

## 5. Conclusion

Our study demonstrated that electroacupuncture on bilateral ST36 acupoints at 15 Hz for 30 minutes and 60 minutes could be an effective means of lowering plasma glucose in a STZ-IDDM rat model during isoflurane anesthesia. We also elucidated a mechanism of action in which EA treatment increases the expression of the IRS-1 and GLUT4 proteins and decreases free fatty acid levels. Further randomized controlled clinical studies are needed to determine whether electroacupuncture is an appropriate adjunct treatment and can be effectively applied to the diabetic patient during surgery.

## Figures and Tables

**Table 1:** Number of Wistar rats in each condition across the experimental treatment (EA vs. non-EA) and group identity (IDDM vs. normal) factors, along with marginal totals.

		Wistar rat group identity		Total
		IDDM	Normal	
Experimental condition	Electroacupuncture	9	4	13
	Nonelectroacupuncture	9	4	13
	Total	18	8	

**Table 2:** Descriptive statistics. Values denote mean (standard deviation) across experimental treatment (EA vs. non-EA), rat group identity (IDDM vs. normal Wistar rats), and time (30 vs. 60 min) for the glucose and FFA measures or protein type (GLUT4 vs. IRS-1).

			Wistar rat group identity	
			IDDM	Normal
Experimental condition	Electroacupuncture	Glucose (relative to baseline)	30 min: −7.690 (9.919) 60 min: −6.744 (8.840)	30 min: −16.626 (11.981) 60 min: −21.476 (23.012)
		FFA (relative to baseline)	30 min: −22.395 (13.507) 60 min: −36.868 (9.754)	30 min: −37.437 (39.982) 60 min: −41.406 (57.908)
		Signal (relative to *β*-actin)	GLUT4: 0.708 (0.259) IRS-1: 1.918 (0.793)	—
	Nonelectroacupuncture	Glucose (relative to baseline)	30 min: 4.919 (7.732) 60 min: 11.331 (17.120)	30 min: 10.244 (15.293) 60 min: 9.050 (13.364)
		FFA (relative to baseline)	30 min: 7.237 (33.351) 60 min: 10.995 (43.578)	30 min: −36.231 (42.170) 60 min: −34.247 (41.181)
		Signal (relative to *β*-actin)	GLUT4: 0.364 (0.173) IRS-1: 0.945 (0.480)	—

## Data Availability

The data used to support the findings of this study are available from the corresponding author upon request.
